# Entropy generation in MHD Casson fluid flow with variable heat conductance and thermal conductivity over non-linear bi-directional stretching surface

**DOI:** 10.1038/s41598-020-69411-2

**Published:** 2020-07-27

**Authors:** Muhammad Sohail, Zahir Shah, Asifa Tassaddiq, Poom Kumam, Prosun Roy

**Affiliations:** 1 Department of Applied Mathematics and Statistics, Institute of Space Technology, P.O. Box 2750, Islamabad, 44000 Pakistan; 2 Center of Excellence in Theoretical and Computational Science (TaCS-CoE), SCL 802 Fixed Point Laboratory, Science Laboratory Building, King Mongkut’s University of Technology Thonburi (KMUTT), 126 Pracha-Uthit Road, Bang Mod, Thrung Khru, Bangkok, 10140 Thailand; 3 Department of Basic Sciences and Humanities, College of Computer and Information Sciences, Majmaah University, Al Majmaah, 11952 Saudi Arabia; 4 KMUTT Fixed Point Research Laboratory, Room SCL 802 Fixed Point Laboratory, Science Laboratory Building, Department of Mathematics, Faculty of Science, King Mongkut’s University of Technology Thonburi (KMUTT), Bangkok, 10140 Thailand; 5 Departments of Medical Research, China Medical University Hospital, China Medical University, Taichung, 40402 Taiwan; 6 Department of Mechanical Engineering, University of Wisconsin, Milwaukee, WI USA

**Keywords:** Mechanical engineering, Fluid dynamics, Applied mathematics

## Abstract

This consideration highlights the belongings of momentum, entropy generation, species and thermal dissemination on boundary layer flow (BLF) of Casson liquid over a linearly elongating surface considering radiation and Joule heating effects significant. Transportation of thermal and species are offered by using the temperature-dependent models of thermal conductivity and mass diffusion coefficient. Arising problem appear in the form of nonlinear partial differential equations (NPDEs) against the conservation laws of mass, momentum, thermal and species transportation. Appropriate renovation transfigures the demonstrated problem into ordinary differential equations. Numerical solutions of renovated boundary layer ordinary differential equations (ODEs) are attained by a proficient and reliable technique namely optimal homotopy analysis method (OHAM). A graphical and tabular interpretation is given for convergence of analytic solutions through error table and flow behavior of convoluted physical parameters on calculated solutions are presented and explicated in this examination. Reliability and effectiveness of the anticipated algorithm is established by comparing the results of present contemplation as a limiting case of available work, and it is found to be in excellent settlement. Decline in fluid velocity and enhancement in thermal and species transportation is recorded against the fluctuating values of Hartman number. Also reverse comportment of Prandtl number and radiation parameter is portrayed. Moreover, it is conveyed that supplementing values of the magnetic parameter condenses the fluid velocity and upsurges the thermal and concentration distributions. Negative impact of elevating Joule heating phenomenon is noted on the molecular stability of the system via Brinkman number $\left( {Br} \right).$ Furthermore, the system’s stability at a molecular level is controlled by diminishing values of radiation $\left( R \right),$ temperature difference $\left( { \in_{1} } \right),$ concentration difference $\left( { \in_{2} } \right),$ diffusion parameters $\left( { \in_{3} } \right)$ and Brinkman number $\left( {Br} \right).$

## Introduction

Stretched flows have much applications in different industrial phenomenon. Time dependent boundary layer flow streaming past a shrinking surface with influence of transverse magnetic field was premeditated by Merkin and Kumaran^1^. They showed that the fluid seemed to be effected by the range of magnetic parameter and dimensionless time parameter, such that for magnetic parameter less than 1, steady state was achieved but for times very large but for magnetic parameter taken as 1, boundary layer flow was achieved for all values of time and the wideness of boundary layer was observed to increase with time. Shehzad et al.^2^ premeditated the contribution of temperature dependent thermal conductivity on Oldroyd-B model past over a stretched surface. Thermal distribution with heat generation on viscoelastic model produced due to the stretching of the sheet was reported by Khan et al.^3^. They have offered the demeanor of several embryonic parameters on velocity field and temperature profile through graphs and tables. They presented the comparative study too in order to discourse the authenticity of the archived solutions. Moreover, they recorded that positive mounting values of heat generation factor serves to enhance the temperature distribution. Khan et al.^4^ dissected the heat and mass transference in a viscous fluid past over a nonlinear lengthening sheet. For comparative study, they offered the numerical and analytical solutions both in their contribution. They found excellent settlement in both the computed solutions. Natural convection flow of copper–water based nanofluid through oddly shaped geometry was studied by Parvin and Chamkha^5^ using penalty finite element method with Galerkin weighted residual technique. The study highlighted the importance of Rayleigh number as with larger value of Rayleigh number the contribution of heat transfers in entropy generation rose while the contribution of viscous dissipation dropped. Hence, with the right range of Rayleigh number, it was believed that stability could be maximized for this particular physical system. Time dependent MHD Casson fluid free flow over an oscillating vertical plate covered with porous media was analyzed by Khalid et al.^6^ using Laplace transform. The results shined light on reduced velocity and risen skin friction on the surface with elevating magnetic parameter. Rauf et al.^7^ investigated boundary layer flow (BLF) of Casson nanofluid (CN) on a sheet elongating in two directions exposed to transverse magnetic field and thermal radiation with mixed convection conditions using Range-Kutta-Fehlberg (RKF45) technique. It was noticed that the system improved thermally with higher radiation. Zaib et al.^8^ concentrated on Casson fluid with effects of viscous dissipation flowing on an exponentially shrinking sheet. The mathematical system was solved numerically using shooting method and dual solutions were obtained for velocity and temperature. The analysis showed a decline in system’s temperature with rising Prandtl number whereas system heated up for enhanced effects of viscous dissipation. Raju et al.^9^ compared Casson fluid with Newtonian fluid over an exponentially broadening surface under the effects of thermal radiation, viscous dissipation and magnetism. Using MATLAB bvp4c package, the results showed an improvement in the system thermally for higher viscous dissipation effects and that heat transfer rates were better for Casson fluid compared to Newtonian fluid. An almost similar study done by Soluchana et al^10^ in which three dimensional Casson nanofluid is compared to Newtonian fluid using RK shooting technique. The study incorporated MHD effects and the fluid is passed over a stretching plane. Both mass and heat transfer rates boosted for larger stretching ratio parameter and here again Casson fluid had better heat, and mass, transfer rates compared to Newtonian fluid. Kumaran and Sandeep^11^ worked on the comparison of MHD Casson and Williamson fluid streaming on top of an upper paraboloid of revolution taking into account the thermophoresis and Brownian motion impacts. Using the RK method with shooting technique, Casson fluid turned out to be better than Williamson fluid in terms of heat and mass transfer. MHD Casson fluid streaming past a wedge with influence of binary chemical reactions and activation energy was premeditated by Zaib et al.^12^. With the use of modified Arrhenius function to represent activation energy the model was developed and solved using keller box method. The results highlighted decaying temperature and concentration boundary layers and enhanced fluid stream velocity for elevating Casson fluid parameter. Irfan et al.^13^ analyzed the impact of stretching rate ratio parameter on the three dimensional forced convection Carreau nanofluid flow over a surface stretching in two directions keeping variable thermal conduction and heat generation/absorption as significant effects. After solving the equations using MATLAB bvp4c package, it was seen that elevated stretching rate ratio caused a drop in skin friction coefficient in both $x$ and $y$ directions for shear thickening as well as thinning cases of the Carreau fluid. MHD mixed convection Casson fluid flow with effects of double stratification and heat cohort/immersion was considered by Rehman et al.^14^. The study proceeded with the use of RK method with shooting technique and the results showed a deescalating velocity in regard with rising Casson fluid parameter. Merkin et al.^15^ worked on stagnation point flow over an exponentially elongating/dwindling cylinder using shooting method. It was seen that the solution came out to be unique for the stretching case, whereas dual solutions appeared for the shrinking case further developing the study based on the critical values defined through patterns observed. Stagnation point nanofluid flow streaming on top of a stretching surface was investigated by Jalilpour et al.^16^ using the RK-4 numerical technique considering the impact of thermal radiation. Higher thermophoresis brought about a negative change in the Nusselt number while a rise in the mass transfer rate, while radiation boosted the Sherwood number. Sohail et al.^17^ studied the second grade fluid on a stretching surface using HAM. Considerations of generalized Fourier’s and Fick’s laws were taken to incorporate the Cattaneo-Christov heat and mass flux in the physical system. It was seen that compared to non-Fick’s and non-Fourier’s laws, temperature and concentrations was higher for classical Fick’s and Fourier’s laws. Zaib et al.^18^ emphasized on stagnation point flow of Casson nanofluid on top of a plate immersed in Darcy-Brinkman porous medium. Non-linear radiation and activation energy was incorporated using modified Arrhenius function and impact of binary chemical reaction was also considered. With the use of shooting method nanoparticles concentration became dense for rising influence of activation energy while opposite happened for larger reaction rate. Abrar et al.^19^ studied the entropy generation in a water based titanium dioxide nanofluid that was transported through cilia. Viscous dissipation, radiation and MHD effects were considered significant. Similarly, Rashidi et al.^20^ considered MHD third grade fluid streaming past a sheet stretching in a linear manner and worked on the system using optimal homotopy analysis method (OHAM). Both the studies showed magnetic field and total entropy generation were in direct relation. Raju and Sandeep^21^ worked on Casson nanofluid considered to be flowing on a rotating cone present in a rotating frame using the Range-Kutta (RK) shooting technique. Their results highlighted escalating drag force on the surface and heat transfer rates for higher volume fraction of nanoparticles present in the fluid. Non-Newtonian fluids have been a strong focus of researchers due to their vast properties compared to simple Newtonian fluids. These fluids have a non-linear relation of shear stress to shear strain. Examples of non-Newtonian fluids are numerous, some including: ketchup, honey, blood, molten polymers, clays, paints, etc. Some materialistic characteristics are reported in^22^. Casson fluid is a non-Newtonian fluid that best describes fluids like blood, honey, jelly, etc., where fluids behave like elastic solids and have molecular chains connecting the particles within. This fluid model is better at describing the rheological properties of such fluids that cannot be described using viscoelastic fluid models. Casson fluid is a shear thinning fluid liquid with three assumptions: (i) at zero shear rate the viscosity is infinite, (ii) no flow exists below the yield stress and (iii) at infinite shear the viscosity is neglible as explicated by Reddy et al.^23^. Unsteady Casson fluid streaming on top of a enlarging sheet was investigated by Khan et al.^24^ using HAM. They noticed that the thickness of the boundary layer for unsteady case was very less compared to the steady case. Sheikholeslami et al.^25^ worked on an unsteady MHD radiative nanofluid flowing over an oscillating vertical plate with effects of heat generation and absorption taken into account. Flow stream velocity was seen to decline with heightening magnetic encouragement. Zaib et al.^26^ emphasized on the stability of stagnation point flow of Williamson nanofluid flowing on top of a moving plate through entropy generation. Impacts of activation energy and binary chemical reactions were taken into consideration, where activation energy was incorporated using modified Arrhenius function. The mathematical system was analyzed numerically using shooting method and the results portrayed heightened concentration boundary layer for large activation energy whereas the opposite was noted for increased reaction rates. Moreover, entropy generation for Williamson fluid was greater in comparison to Newtonian fluid. Usman et al.^27^ premeditated the impression of non-linear thermal radiation and thermal conductivity depending on time on the rotating flow of hybrid copper–aluminum oxide nanofluid with water as base fluid. The fluid flowed on three dimensional stretching surface. Using least square method (LSM) to solve the mathematical system it was found that the hybrid nanofluid had lower thermal advantages compared to individual copper and aluminum oxide nanofluids. Steady axisymmetric rotational stagnation point flow was analyzed by Lok et al.^28^ using the bvp4c MATLAB package over a rotating permeable shrinking/stretching disk. The study concluded the existence of three solutions depending on the stretching rate, the rotation rate and the fluid withdrawal/injection. Reddy et al.^29^ compared the Casson and Maxwell MHD fluids over a stretched surface using the numerical scheme RK Fehlberg. The result highlighted higher mass transfer rates but reduced heat transfer rates for Maxwell fluid in comparison to Casson fluid. Kumar et al.^30^ considered MHD Casson and Maxwell fluid with effects of chemical reaction and heat generation/absorption. With the use of RK method along with shooting technique comparison between both the fluids was studied. The results highlighted reduced heat and mass transfer rates for heightened magnetic influence for both Casson as well as Maxwell fluid. Mehryan et al.^31^ considered using periodic magnetic field on the natural convection flow of ${Fe}_{3}{O}_{4}$-water based nanofluid within a square enclosed system to study the entropy generation. With the help of Galerkin finite element method, for values of Hartmann number less than 10, the presence of nanoparticles seemed to boost entropy generation, while for values greater than 10, nanoparticles’ influence positively impacted the stability of the molecules within the system. Hamid et al.^32^ studied the characteristics of variable thermal conductivity on mixed convective radiative flow of rotating nanofluid past over an elongating sheet which is stretched horizontally. They presented the finite element analysis of the modelled physical system. They have mentioned that brick shape nanoparticles can work as a coolant for the thermal system. Legendre wavelets approach was presented by Soomro et al.^33^ to investigate the influence of thermal and momentum slips on mixed convective Williamson model. They concluded that augmenting values of Prandtl number increases the heat transfer rate and mass transport rate is assisted due to increment in Lewis number. Shah et al.^34^ emphasized on the Casson micropolar ferrofluid flowing on a linearly stretching sheet taking transverse magnetohydrodynamic (MHD) and thermal radiation effects into contemplation. Homotopy analysis method (HAM) was used to solve the mathematical system. The results concluded a significant rise in the surface drag force with large magnetic influence and higher heat transfer rates with upsurge in Prandtl values. Hamid et al.^35^ considered a stretching sheet covered with MHD Casson fluid influenced by linearly acting thermal radiation. Dual solutions were calculated and was seen that both upper and lower profiles of the temperature were an increasing function of radiation. MHD nanofluid flowing through an enclosed area filled with minor pores was investigated by Sheikholeslami^36^. A new numerical technique called the control volume finite element process was used to solve the mathematical system and the results shows a downfall in the heat transfer rates with rising magnetic influence. Ullah et al.^37^ studied the MHD squeezing viscous fluid flowing between two parallel plates flooded with minor pores. The focus was on the comparison of numerous schemes like optimal homotopy analysis method (OHAM), homotopy perturbation (HP), differential transform (DT), Daftardar Jafari (DJ) and Adomian decomposition method (ADM). In view of the results, they recorded that OHAM and HPM were more accurate. Heat transfer rates were studied for natural convection flow of Casson fluid flowing through a partially heated trapezoidal cavity using Galerkin finite element method (GFEM) by Hamid et al.^38^. Through results it was seen by elevating Casson fluid parameter enhanced heat transfer rates in the middle of the cavity. Sheikholeslami et al.^39^ analyzed the Nusselt number for alumina nanofluid streaming between two parallel plates, top plate being permeable, using neural networks attaining the numerical data by RK method. Nusselt number was positively influenced by the nanofluid concentration. Hosseini et al.^40^ premeditated the belongings of using aluminum oxide water based nanofluid paired with symmetric heating and MHD on the entropy generation in the porous horizontal channel. Irreversibility of fluid heat transfer under the influence of MHD were less than when MHD impact was considered negligible. Hamid et al.^41^ used the finite difference approach to captures the features of slip flow of Prandtl model with mass and heat transportation under thermos-diffusion effects. They commented that unsteadiness parameter upsurges the velocity, mass and temperature distribution profiles. MHD flow in a cavity with radiative heat transportation and mass transfer was explored by Usman et al.^42^. They presented that radiation parameter serves as a coolant for the thermal system which is much needed because higher warming effects can cause a failure for the thermal system. Numerically the flow of Carreau fluid with heat and mass transportation induced due to the stretching of the sheet was surveyed by Ali et al.^43^. They have shown that Lorentz force cause to boost the temperature field and lessen the velocity profile. Ali et al.^44^ discussed the features of chemically reactive species with thermal distribution in a Jeffery liquid past over a stretching cylinder. Generalized model for heat flux is presented to model the energy equation. Contribution of heat and mass in a yield exhibiting fluid model in a cylinder was explored by Ali et al.^45^. Revised definition for heat flux model is used with heat generation. They have shown that curvature parameter and Lorentz force shows opposite impact of flow velocity. Mixed convective flow of Newtonian liquid in a stretched cylinder with heat and mass transfer was studied by Ali et al.^46^. Significant support of joule heating and heat cohort are encompassed in their study. Ali et al.^47^ discussed the flow past over a rotating disk with heat transportation. They presented the involvement of homogeneous-heterogeneous reaction in their worthy investigation.

Comprehensive cited literature survey fails to report the irreversibility analysis of Casson fluid obeying temperature dependent conductance and thermal conductivity. Over the past years, MHD Casson fluid under the inspiration of thermal radiation, temperature dependent conductivity and temperature dependent mass diffusivity over a linearly elongating surface has not been considered. Moreover, investigation of entropy generation for such scenario is new as well which plays a vital role in numerous industrial disciplines. This study aims to focus on the considered phenomenon with the help of optimal homotopy analysis method (OHAM)^48–54^ and obtain graphical representations for the velocity, temperature, concentration changes and molecular stability of the system. Comparative study is also presented in order to verify the effectiveness of the anticipated computational tool. This exploration is organized as follows: literature survey is presented in “Introduction”, physical happening is listed in “Mathematical drafting (MD) for the physical happening via boundary layer theory (BLT)”, irreversibility phenomenon is listed in “Entropy generation computation (EGC)”, used procedure is mentioned in “Solution via OHAM”, physical interpretation is organized in “Results and discussion” and the last section is “Conclusion”.

## Mathematical drafting (MD) for the physical happening via boundary layer theory (BLT)

**Figure 1:**
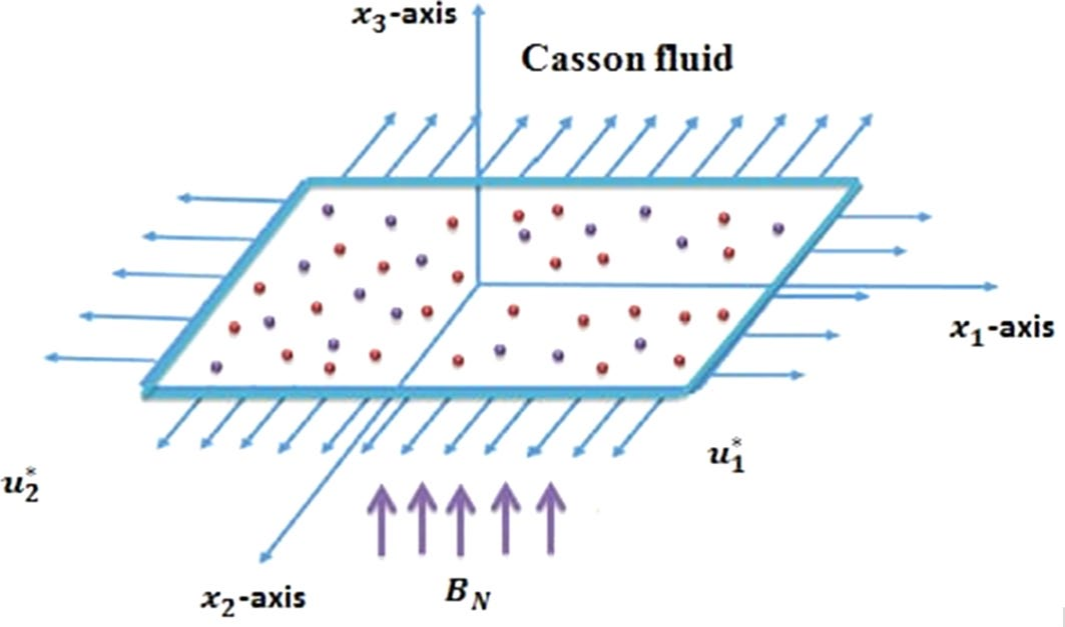
Geometry of the modeled physical problem with Coordinate system.

Magnetohydrodynamic boundary layer flow (MBLF) of yield stress demonstrating Casson liquid^6–12, 14, 18, 21, 23, 24, 30, 32, 35, 38, 45, 51^ over a linear stretchable surface with the transportation of heat and mass is conveyed in current work. Schematic diagram for the deliberated physical happening is shown through Fig. 1.

Flow is manufactured due to the enlarging of the bi-directional strained surface^4, 49–51^ under constant pressure. In current assessment, Cartesian coordinates system is used to model the conservation laws. Magnetic field ${\mathbf{B}} = \left[ {0,\, \, 0,\, \, B_{N} } \right]$ is tarnished normal to stretchable surface. Attractive Reynolds number is anticipated to be less, so that the predominant magnetic field is deserted associated to pragmatic magnetic field. Heat and mass repositioning contrivances are recognized by exhausting the temperature dependent thermal conductivity^27, 32^ and diffusion coefficient. Moreover, impact of radiation^32, 43^ and Joule heating^46^ is taken in this exploration. With such implication predominant boundary layer equations that entitles the flow state are enunciated as

1 $$\frac{{\partial u_{1}^{*} }}{{\partial x_{1} }} + \frac{{\partial u_{2}^{*} }}{{\partial x_{2} }} + \frac{{\partial u_{3}^{*} }}{{\partial x_{3} }} = 0$$

2 $$u_{1}^{*} \frac{{\partial u_{1}^{*} }}{{\partial x_{1} }} + u_{2}^{*} \frac{{\partial u_{2}^{*} }}{{\partial x_{2} }} + u_{3} \frac{{\partial u_{1}^{*} }}{{\partial x_{3} }} = \nu_{N} \left( {1 + \frac{1}{\beta }} \right)\frac{{\partial^{2} u_{1}^{*} }}{{\partial x_{3}^{2} }} - \frac{{\sigma_{N} B_{N}^{2} }}{{\rho_{N} }}u_{1}^{*}$$

3 $$u_{1}^{*} \frac{{\partial u_{2}^{*} }}{{\partial x_{1} }} + u^{*}_{2} \frac{{\partial u_{2}^{*} }}{{\partial x_{2} }} + u^{*}_{3} \frac{{\partial u_{2}^{*} }}{{\partial x_{3} }} = \nu_{N} \left( {1 + \frac{1}{\beta }} \right)\frac{{\partial^{2} u_{2}^{*} }}{{\partial x_{3}^{2} }} - \frac{{\sigma_{N} B_{N}^{2} }}{{\rho_{N} }}u_{2}^{*}$$

4 $$u_{1}^{*} \frac{{\partial T_{1}^{*} }}{{\partial x_{1} }} + u_{2}^{*} \frac{{\partial T_{1}^{*} }}{{\partial x_{2} }} + u_{3}^{*} \frac{{\partial T_{1}^{*} }}{{\partial x_{3} }} = \frac{1}{{\rho_{N} c_{p} }}\frac{\partial }{{\partial x_{3} }}\left( {K\left( {T_{1}^{*} } \right)\frac{{\partial T_{1}^{*} }}{{\partial x_{3} }}} \right) + \frac{16}{{\rho_{N} c_{p} 3K_{m} }}T_{\infty }^{3} \frac{{\partial^{2} T_{1}^{*} }}{{\partial x_{3}^{2} }} + \frac{{\sigma_{N} B_{N}^{2} }}{{\rho_{N} c_{p} }}\left[ {\left( {u_{1}^{*} } \right)^{2} + \left( {u_{2}^{*} } \right)^{2} } \right],$$

5 $$u_{1}^{*} \frac{{\partial C_{1}^{*} }}{{\partial x_{1} }} + u_{2}^{*} \frac{{\partial C_{1}^{*} }}{{\partial x_{2} }} + u_{3}^{*} \frac{{\partial C_{1}^{*} }}{{\partial x_{3} }} = \frac{\partial }{{\partial x_{3} }}\left( {D\left( {T_{1}^{*} } \right)\frac{{\partial C_{1}^{*} }}{{\partial x_{3} }}} \right),$$

Following boundary conditions appears against the modeled situation recorded in Eqs. (2–5) has been developed via no slip perception

6 $$\left\{ \begin{gathered} u_{1}^{*} = U_{w}^{ * } (x_{1} ) = a_{1}^{ * } x_{1} ,\;u_{2}^{*} = V_{w}^{ * } (x_{2} ) = b_{1}^{ * } x_{2} ,\;u_{3}^{*} = 0,\, \, T_{1}^{*} = T_{w} ,\;C_{1}^{*} = C_{w} \, \;{\text{at }}\;x_{3} = 0, \hfill \\ u_{1}^{*} \to 0,\, \, u_{2}^{*} \to 0,\, \, C_{1}^{*} \to C_{\infty } ,\, \, T_{1}^{*} \to T_{\infty } \;{\text{as }}x_{3} \to \infty . \hfill \\ \end{gathered} \right.$$

## Entropy generation computation (EGC)

Entropy phenomenon^5, 12, 19, 20, 26, 31, 40, 49, 52, 53^ for Casson fluid past over a bidirectional strained surface with heat and mass conveyance having variable thermal conductivity and temperature dependent mass diffusion coefficient is communicated as

7 $$Eg\left( \xi \right) = \frac{{K\left( {T_{1}^{*} } \right)}}{{T_{\infty }^{2} }}\left[ {1 + \frac{{16\sigma_{N} T_{\infty }^{3} }}{{3K_{m} }}} \right]\,\left( {\frac{{\partial T_{1}^{*} }}{{\partial x_{3} }}} \right)^{2} + \frac{{R_{D} }}{{C_{\infty } }}\left( {\frac{{\partial C_{1}^{*} }}{{\partial x_{3} }}} \right)^{2} + \frac{{R_{D} }}{{T_{\infty } }}\left( {\frac{{\partial C_{1}^{*} }}{{\partial x_{3} }}} \right)\,\left( {\frac{{\partial T_{1}^{*} }}{{\partial x_{3} }}} \right) + \frac{{\sigma_{N} }}{{T_{\infty } }}B_{N}^{2} \left[ {\left( {(u_{1}^{*} )^{2} + (u_{2}^{*} )^{2} } \right)} \right]$$

Equation (7) comprehends concentration, Joule heating and thermal irreversibility and its dimensionless relation is articulated as

8 $$Eg\left( \xi \right) = (1 + \varepsilon_{a} \theta + \frac{4}{3}R)\left( {\theta^{\prime}} \right)^{2} \left( { \in_{1} } \right)^{2} + HaBr \in_{1} \left[ {(f^{\prime})^{2} + \left( {g^{\prime}} \right)^{2} } \right] + \in_{3} \in_{2} \left( {\varphi^{\prime}} \right)^{2} + \in_{1} \in_{3} \theta^{\prime}\varphi^{\prime}$$

Essential transmutations are

9 $$\left\{ \begin{gathered} u_{1}^{*} = a_{1}^{ * } x_{1} f^{\prime}(\xi ),\, \, u^{*}_{2} = b_{1}^{ * } x_{2} g^{\prime}(\xi ),\, \, u_{3}^{*} = - \sqrt {a_{1}^{ * } \nu_{N} } (f(\xi ) + g(\xi )), \hfill \\ \xi = \sqrt {\frac{{a_{1}^{ * } }}{{\nu_{N} }}} x_{3} ,\, \, \theta (\xi ) = \frac{{T_{1}^{*} - T_{\infty } }}{{T_{w} - T_{\infty } }},\, \, \varphi (\xi ) = \frac{{C_{1}^{*} - C_{\infty } }}{{C_{w} - C_{\infty } }}. \hfill \\ \end{gathered} \right.$$

Exploiting above declared renovations Eqs. (2–3), (10, 11) and (14) takes the form

10 $$\left( {1 + \frac{1}{\beta }} \right)f^{\prime\prime\prime} - \left( {f^{\prime}} \right)^{2} + (f + g)f^{\prime\prime} - \left( {Ha} \right)^{2} f^{\prime} = 0$$

11 $$\left( {1 + \frac{1}{\beta }} \right)g^{\prime\prime\prime} - \left( {g^{\prime}} \right)^{2} + (f + g)g^{\prime\prime} - \left( {Ha} \right)^{2} g^{\prime} = 0$$

12 $$\frac{1}{\Pr }\left( {1 + \frac{4}{3}R + \varepsilon_{a} \theta } \right)\theta^{\prime\prime} + (f + g)\theta^{\prime} + \left( {Ha} \right)^{2} Ec\left[ {\left( {f^{\prime}} \right)^{2} + \left( {g^{\prime}} \right)^{2} } \right] = 0$$

13 $$\frac{1}{Sc}\left( {1 + \varepsilon_{b} \theta } \right)\varphi^{\prime\prime} + (f + g)\varphi^{\prime} = 0$$

Renovated boundary conditions are

14 $$\left\{ \begin{gathered} f = 0,\, \, f^{\prime} = 1,\, \, g = 0,\, \, g^{\prime} = \alpha ,\, \, \theta = 1,\,\;\varphi = 1\;{\text{at}}\;\xi = 0, \hfill \\ f^{\prime} \to 0,\, \, g^{\prime} \to 0,\, \, \theta \to 0,\, \, \varphi \to 0\;{\text{as }}\xi \to \infty . \hfill \\ \end{gathered} \right.$$

All dimensionalized variables and parameters are shown in Table 1.

**Table 1:** Nomenclature.

$$u_{1}^{*} ,$$$$u_{2}^{*}$$ and $$u_{3}^{*}$$	Velocity components	$$x_{1} - ,$$$$x_{2} -$$ and $$x_{3} -$$	Space coordinates
$\left( \beta \right) = \frac{{\mu_{\delta } \sqrt {2\pi_{\delta } } }}{{p_{\delta } }}$	FLUID parameter	$\left( {\nu_{N} } \right)$	Kinematic viscosity
$\left( {\sigma_{N} } \right)$	ELECTRICAL conductivity	$\left( {B_{N} } \right)$	Magnetic field strength
$\left( {\rho_{N} } \right)$	Fluid density	$\left( {T_{1}^{*} } \right)$ and $\left( {C_{1}^{*} } \right)$	Fluid temperature and concentration
$\left( {c_{p} } \right)$	Specific heat	$\left( {K_{m} } \right)$	Mean absorption coefficient
$K\left( {T^{*}_{1} } \right) = 1 + \varepsilon_{a} \theta$	Temperature dependent thermal conductivity	$D\left( {T_{1}^{*} } \right) = 1 + \varepsilon_{b} \theta$	Temperature dependent diffusion coefficient
$\left( {Ha} \right)^{2} = \frac{{\left( {\sigma_{N} } \right)\left( {B_{N} } \right)^{2} }}{{\left( {\rho_{N} } \right)a_{1}^{ * } }}$	Magnetic parameter	$\left( {\Pr } \right) = \frac{{\left( {\nu_{N} } \right)}}{{\delta^{ * } }}$	Prandtl number
$\left( {Sc} \right) = \frac{{D_{\beta } }}{{\delta^{ * } }}$	Schmidt number	$\xi = \sqrt {\frac{{a_{1}^{ * } }}{{\nu_{N} }}} x_{3}$	Dimensionless independent variable
$\left( \alpha \right) = \frac{{b_{1}^{ * } }}{{a_{1}^{ * } }}$	Ratio parameter	$\left( {Ec} \right) = \frac{{U_{w}^{2} \mu_{F} }}{{T_{w} - T_{\infty } }}$	Eckert number
$\left( {Br} \right)$	Brinkman number	$\left( { \in_{1} } \right)$	Temperature difference parameter
$\left( R \right) = \frac{4}{{3kK_{m} }}\left( {\sigma_{N} } \right)T_{\infty }^{3}$	Radiation parameter	$\left( { \in_{2} } \right)$ and $\left( { \in_{3} } \right)$	Concentration difference and diffusion parameter

## Solution via OHAM

Due to a couple nonlinear system for controlling differential equations system it is recommended to optimize homotopy scheme for calculating the solutions. The proposed scheme is parameter free or large and does not have to be set aside. This method has no stability issues as seen in numerical schemes. It can be applied to infinite domains and can address linear, non-linear, homogeneous and inhuman problems in a similar way. This scheme requires selecting a linear operator and initial guess. An initial guess is chosen in a way that satisfies the given boundary conditions. Correct selection of initial guesses guarantees convergence of the homotopic procedure.

Initial guesses $\left( {f_{i}^{ * } (\xi )\, \, g_{i}^{ * } (\xi )\, \, \theta_{i}^{ * } (\xi )\, \, \varphi_{i}^{ * } (\xi )} \right)$ with operators $\left( {\pounds_{f}^{ * } ,\, \, \pounds_{g}^{ * } \, \, \pounds_{\theta }^{ * } \, \, \pounds_{\varphi }^{ * } } \right)$ are listed below

15 $$f_{i}^{ * } (\xi ) = 1 - \frac{1}{{e^{\xi } }},\, \, g_{i}^{ * } (\xi ) = \alpha (1 - \frac{1}{{e^{\xi } }}),\, \, \theta_{i}^{ * } (\xi ) = \frac{1}{{e^{\xi } }},\, \, \varphi_{i}^{ * } (\xi ) = \frac{1}{{e^{\xi } }}$$

16 $$\pounds_{f}^{ * } = \frac{{\mathbf{\mathbb{Z}}}}{{{\mathbf{\mathbb{Z}}}\xi }}\left( {\frac{{{\mathbf{\mathbb{Z}}}^{2} }}{{{\mathbf{\mathbb{Z}}}\xi^{2} }} - 1} \right)\,f,\, \, \pounds_{g}^{ * } = \frac{{\mathbf{\mathbb{Z}}}}{{{\mathbf{\mathbb{Z}}}\xi }}\left( {\frac{{{\mathbf{\mathbb{Z}}}^{2} }}{{{\mathbf{\mathbb{Z}}}\xi^{2} }} - 1} \right)\,g,\, \, \pounds_{\theta }^{ * } = \left( {\frac{{{\mathbf{\mathbb{Z}}}^{2} }}{{{\mathbf{\mathbb{Z}}}\xi^{2} }} - 1} \right)\,\theta ,\, \, \pounds_{\varphi }^{ * } = \left( {\frac{{{\mathbf{\mathbb{Z}}}^{2} }}{{{\mathbf{\mathbb{Z}}}\xi^{2} }} - 1} \right)\,\varphi$$

these operators imitate following characteristic

17 $$\left\{ \begin{gathered} \pounds_{f}^{ * } \left[ {a_{1}^{ * * } + a_{2}^{ * * } e^{\xi } + a_{3}^{ * * } \frac{1}{{e^{\xi } }}} \right] = 0,\, \, \pounds_{g}^{ * } \left[ {a_{4}^{ * * } + a_{5}^{ * * } e^{\xi } + a_{6}^{ * * } \frac{1}{{e^{\xi } }}} \right] = 0 \hfill \\ \pounds_{\theta }^{ * } \left[ {a_{7}^{ * * } e^{\xi } + a_{8}^{ * * } \frac{1}{{e^{\xi } }}} \right] = 0,\, \, \pounds_{\varphi }^{ * } \left[ {a_{9}^{ * * } e^{\xi } + a_{10}^{ * * } \frac{1}{{e^{\xi } }}} \right] = 0 \hfill \\ \end{gathered} \right.$$

where $a_{s}^{ * * }$ $\left( {s = 1 - 10} \right)$ are indiscriminate constants.

### Consequences and argument

This subclass contains graphical results for the convergence of homotopic solutions, dimensionless velocity, temperature and concentration. For this exam, we have set some muddled parameters and change one parameter and then figured out its bearing.

### Convergence analysis

This section contains tabular results in the form of error analysis for the convergence of expected homotopic solutions for dimensionless velocities, temperature and concentration solutions. For this scrutiny we have set some thin parameters and change one parameter and then pressed its behavior. The fixed values are $\left( {Ha = 0.7} \right),$ $\left( {\Pr = 1.0} \right),$ $\left( {Sc = 0.85} \right),$ $\left( {R = 0.24} \right),$ $\left( {Br = 0.7} \right),$ $\left( { \in_{1} = 0.2} \right),$ $\left( { \in_{2} = 0.28} \right)$ and $\left( { \in_{3} = 0.3} \right).$ Convergence of desired solution is depicted in Table 2 which confirms that by increasing order of approximations, error diminishes which collaterals the convergence of suggested scheme. This table presents the direct relation between the errors and approximation order. Higher order of approximation reduces the errors in obtained solutions.

**Table 2:** Convergence examination through error reduction via OHAM.

${s}^{*}$	$\dddot E_{{s^{*} }}^{f} x_{1}$	$\dddot E_{{s^{*} }}^{g}$	$\dddot E_{{s^{*} }}^{\theta }$	$\dddot E_{{s^{*} }}^{\varphi }$	CPU time (s)
$2$	$293593\times {10}^{-6}$	$2.93593\times {10}^{-6}$	$0.000244616$	$0.0000214515$	2.5467
$4$	$1.85543\times {10}^{-8}$	$1.85543\times {10}^{-8}$	$0.0000187684$	$1.03026 \times {10}^{-6}$	10.0456
$6$	3.31176 $\times {10}^{-10}$	3.31176 $\times\,{10}^{-10}$	3.41302 $\times\,{10}^{-6}$	2.51364 $\times {10}^{-8}$	15.6537
$8$	8.81889 $\times {10}^{-12}$	8.81889 $\times\,{10}^{-12}$	5.40688 $\times\,{10}^{-7}$	1.20006 $\times {10}^{-9}$	28.1432
$10$	2.86855 $\times {10}^{-13}$	2.86855 $\times\,{10}^{-13}$	1.14876 $\times\,{10}^{-7}$	5.96324 $\times\,{10}^{-11}$	48.9438
$12$	1.03859 $\times\,{10}^{-14}$	1.03859 $\times\,{10}^{-14}$	2.9143 $\times\,{10}^{-8}$	2.29532 $\times\,{10}^{-12}$	126.2504
$16$	$1.56777\times {10}^{-16}$	1.56777 $\times\,{10}^{-16}$	1.72687 $\times\,{10}^{-9}$	6.03542 $\times\,{10}^{-13}$	354.5284
$20$	$2.53601\times {10}^{-20}$	2.53601 $\times\,{10}^{-20}$	1.17993 $\times\,{10}^{-10}$	2.24072 $\times\,{10}^{-17}$	528.2963

## Results and discussion

This section contains the graphical results for the fluid velocity, temperature and concentration fields against the various influential variables which appears in the transformed system of ordinary differential equations. The impacts of $K\left( {T^{*}_{1} } \right) = 1 + \varepsilon_{a} \theta$ the temperature dependent thermal conductivity, temperature dependent diffusion coefficient $D\left( {T_{1}^{*} } \right) = 1 + \varepsilon_{b} \theta ,$ the magnetic parameter $\left( {Ha} \right),$ the Prandtl number $\left( {\Pr } \right),$ the Schmidt number $\left( {Sc} \right),$ the radiation parameter $\left( R \right),$ the Brinkman number $\left( {Br} \right),$ temperature difference parameter $\left( { \in_{1} } \right),$ concentration difference parameter $\left( { \in_{2} } \right)$ and the diffusion parameter $\left( { \in_{3} } \right)$ are noted on momentum, thermal, mass boundary layers and entropy generation through graphical representations.

**Figure 2:**
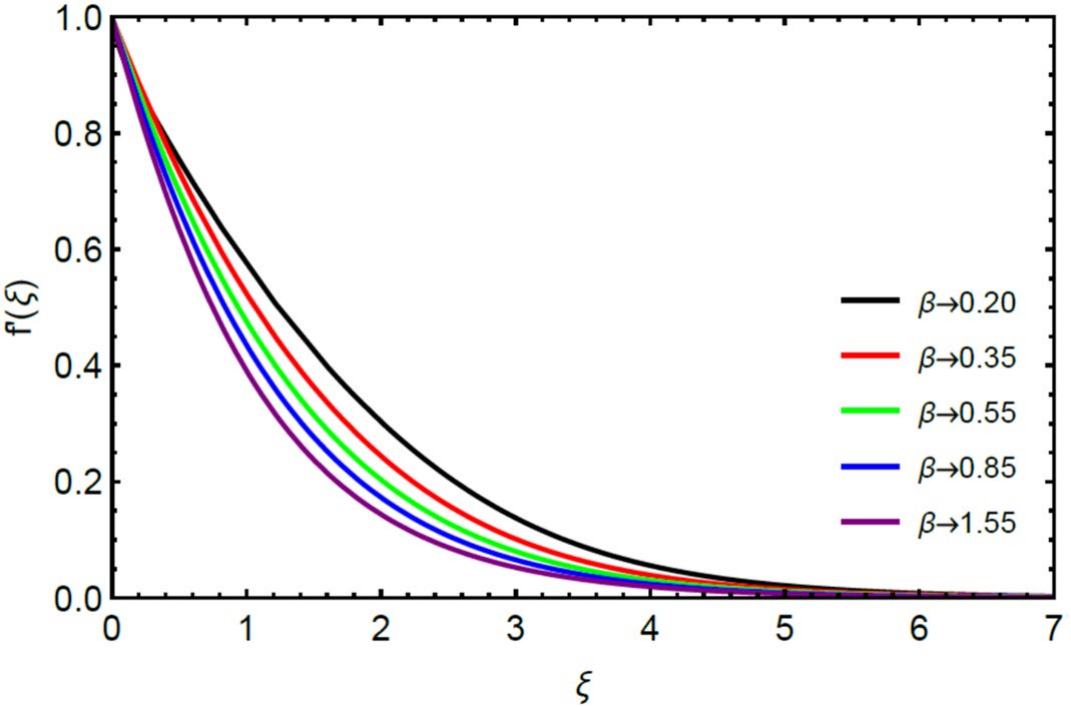
Inspiration of $\left( \beta \right)$ on $f^{\prime}\left( \xi \right)$.

**Figure 3:**
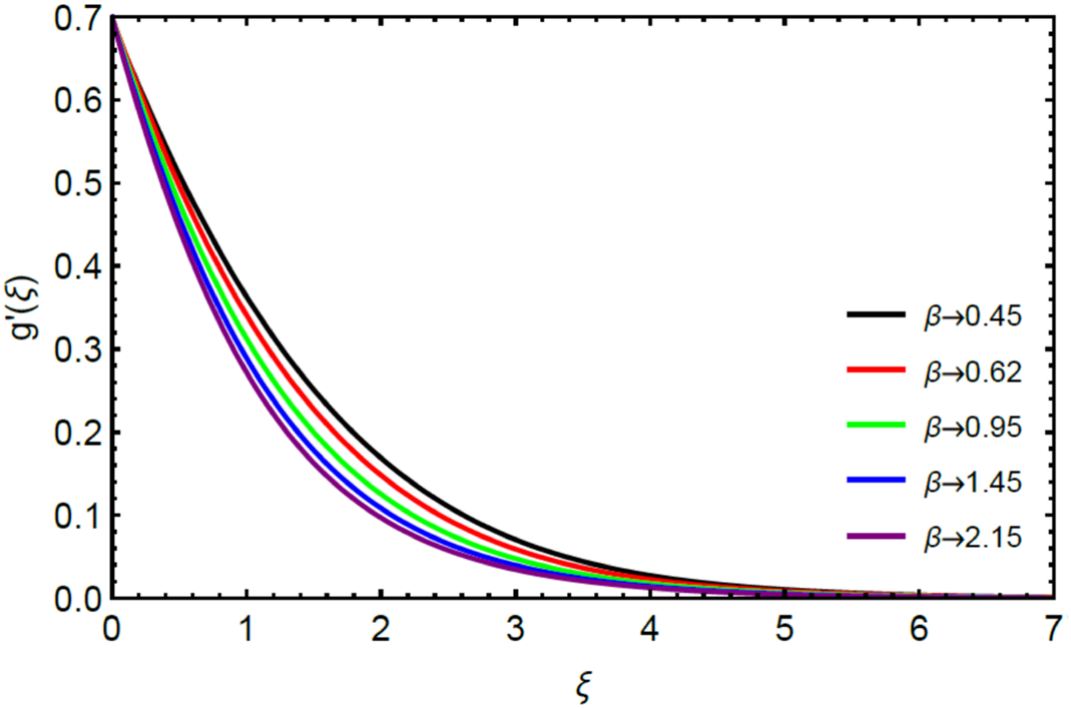
Influence of $\left( \beta \right)$ on $g^{\prime}\left( \xi \right)$.

**Figure 4:**
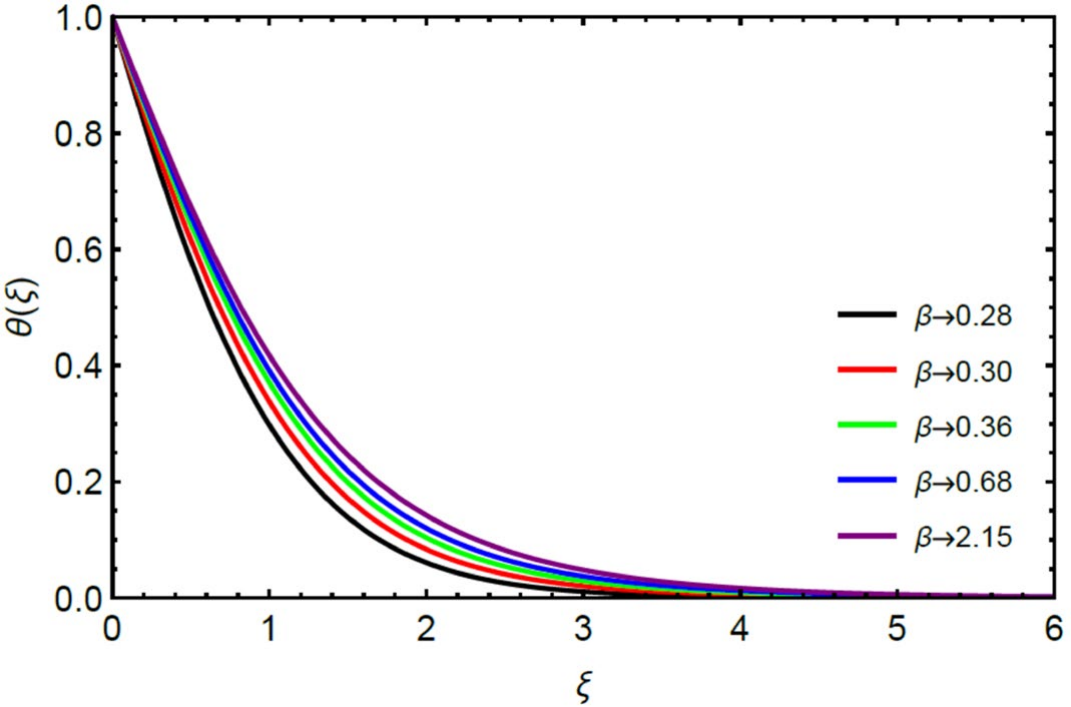
Comportment of $\left( \beta \right)$ on $\theta \left( \xi \right)$.

**Figure 5:**
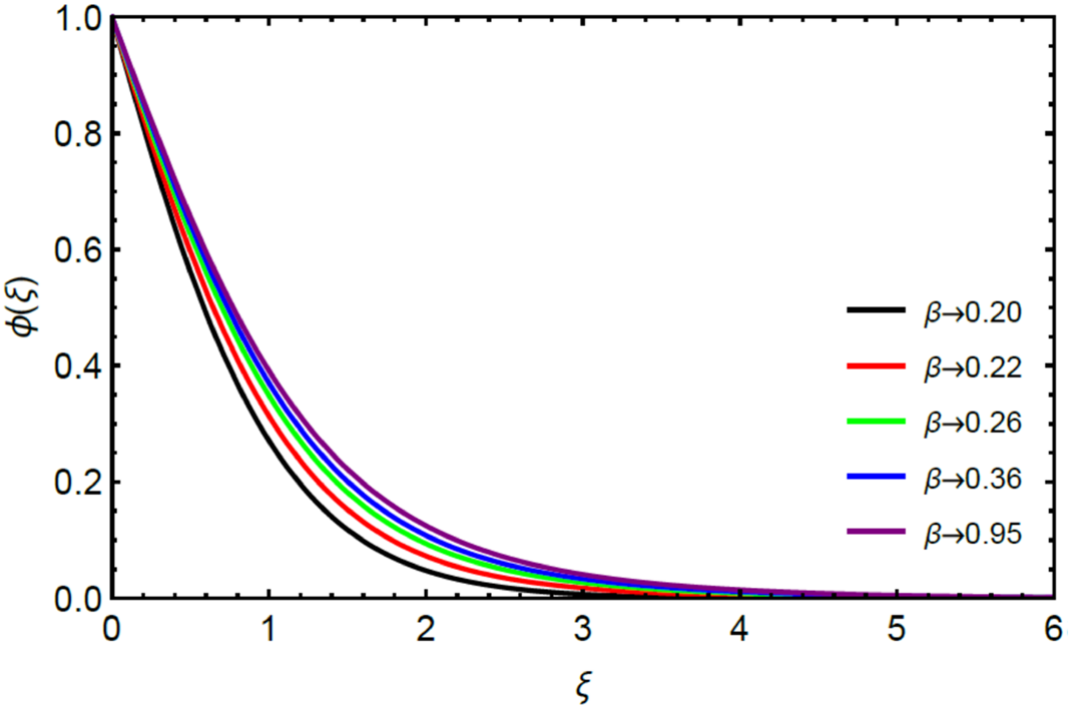
Bearing of $\left( \beta \right)$ on $\phi \left( \xi \right)$.

**Figure 6:**
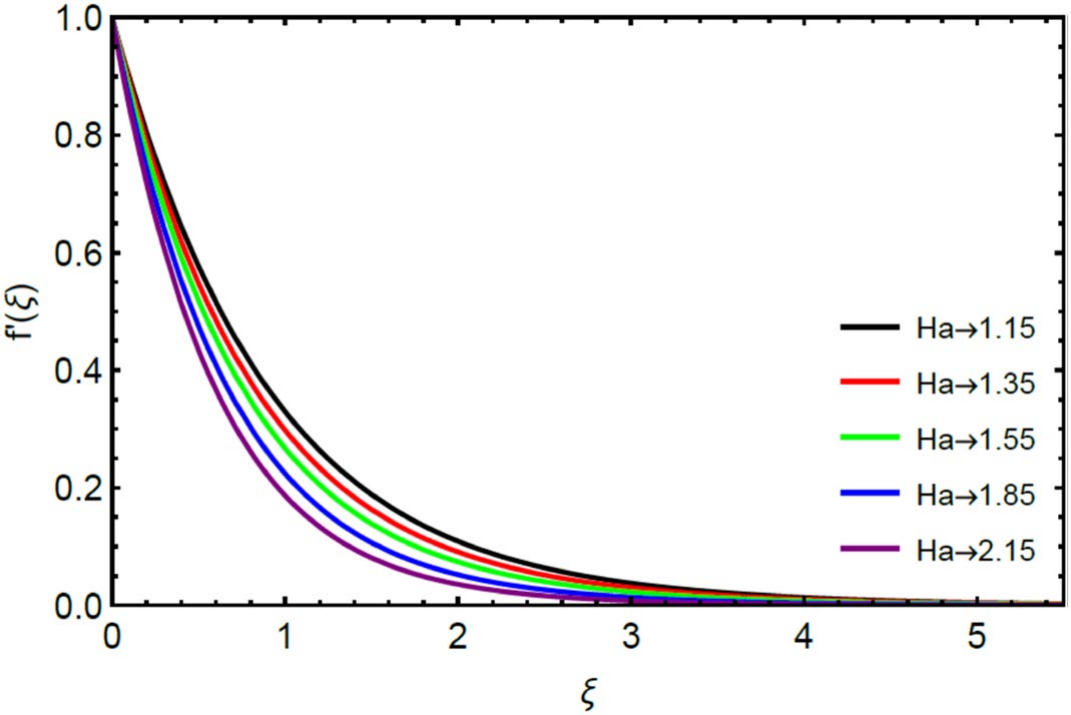
Effect of $\left( {Ha} \right)$ on $f^{\prime}\left( \xi \right)$.

**Figure 7:**
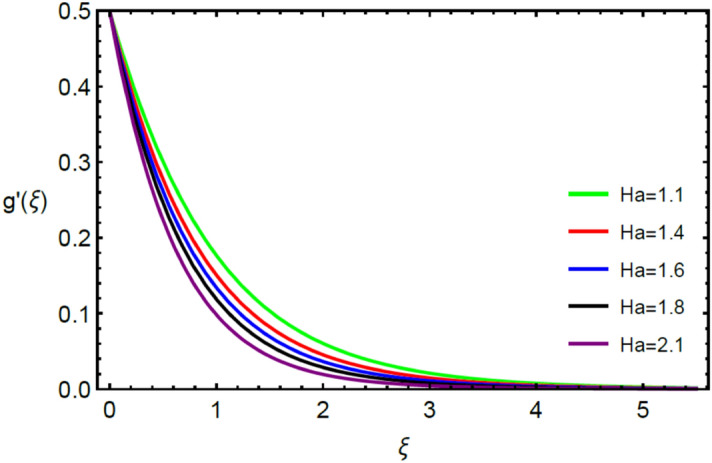
Inspiration of $\left( {Ha} \right)$ on $g^{\prime}\left( \xi \right)$.

**Figure 8:**
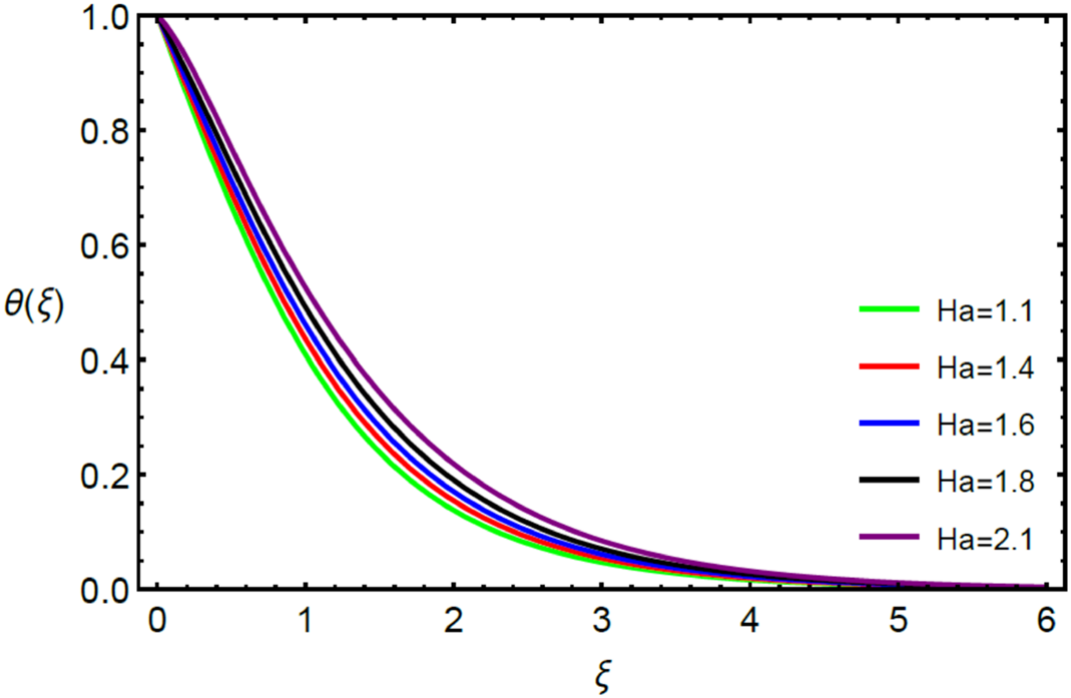
Encouragement of $\left( {Ha} \right)$ on $\theta \left( \xi \right)$.

**Figure 9:**
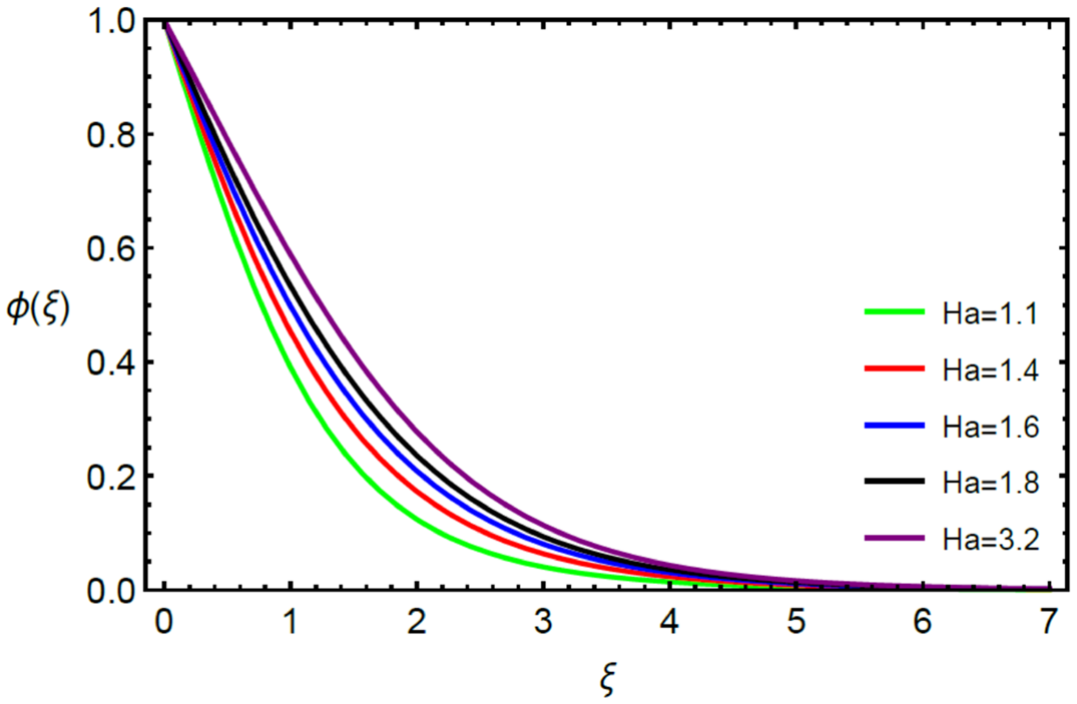
Bearing of $\left( {Ha} \right)$ on $\phi \left( \xi \right)$.

**Figure 10:**
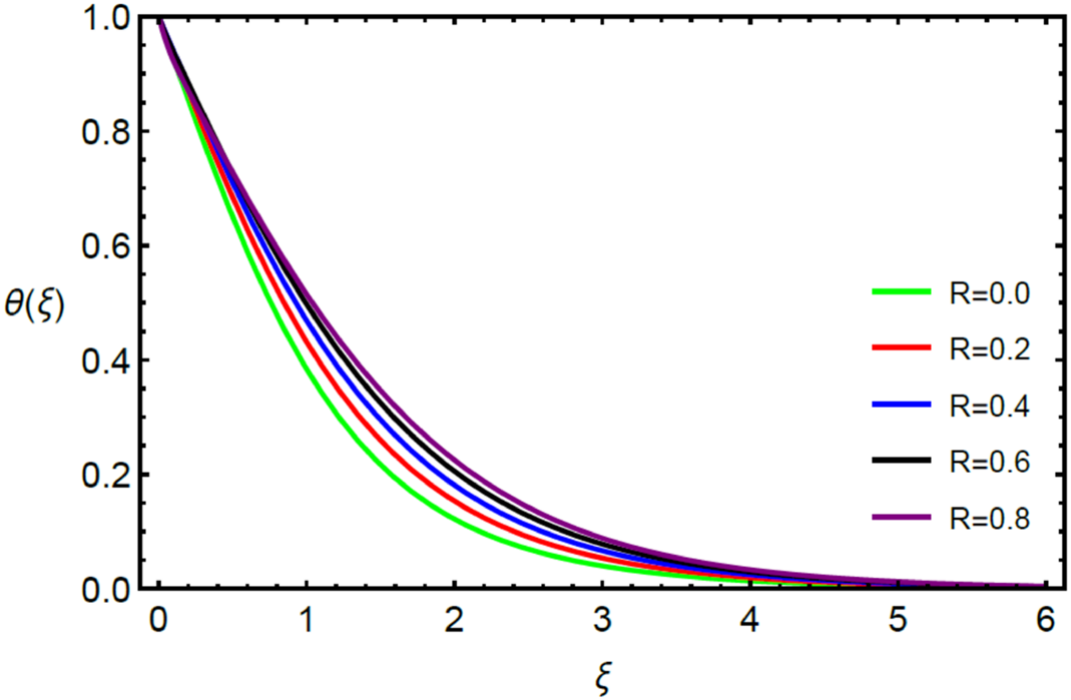
Comportment of $\left( R \right)$ on $\theta \left( \xi \right)$.

**Figure 11:**
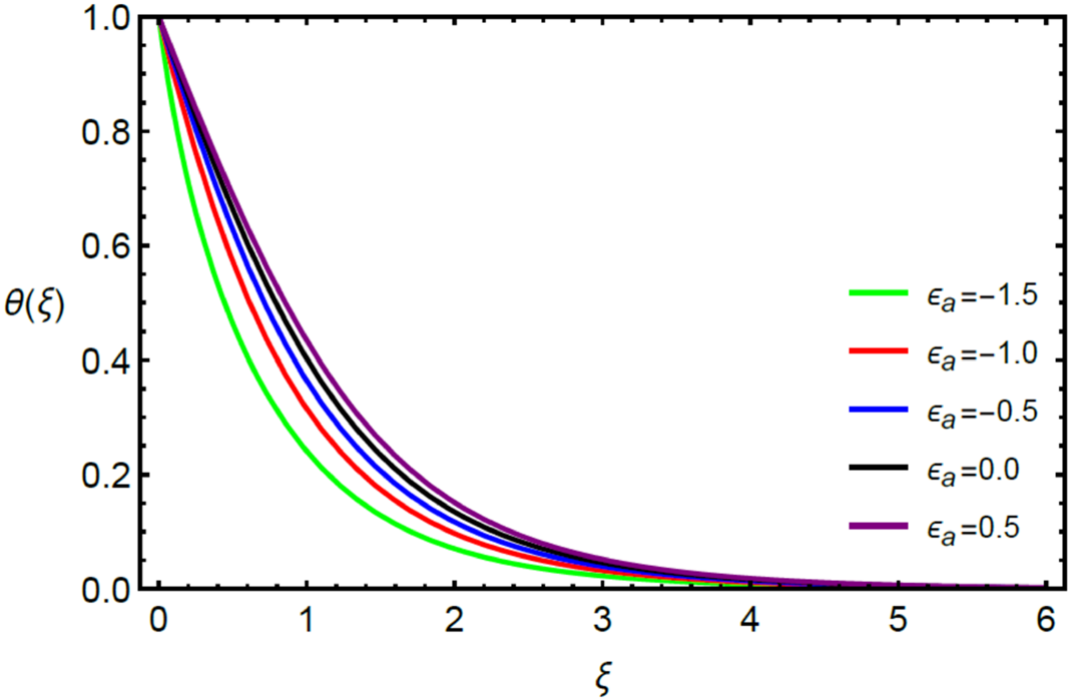
Involvement of $\left( { \in_{a} } \right)$ against $\theta \left( \xi \right)$.

**Figure 12:**
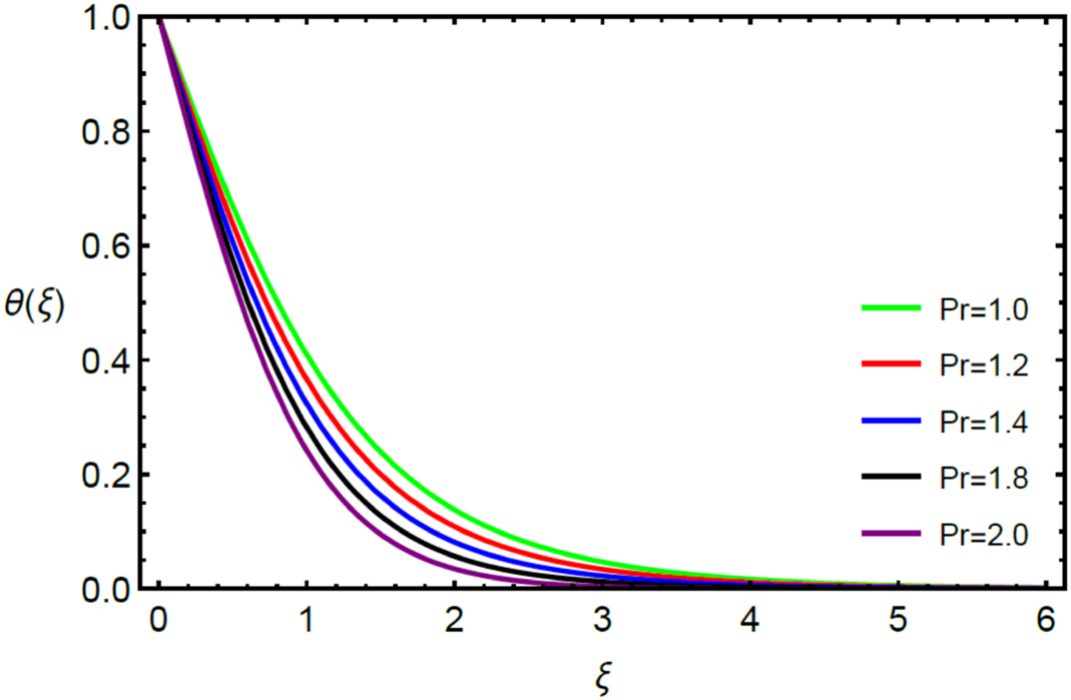
Influence of $\left( {\Pr } \right)$ on $\theta \left( \xi \right)$.

**Figure 13:**
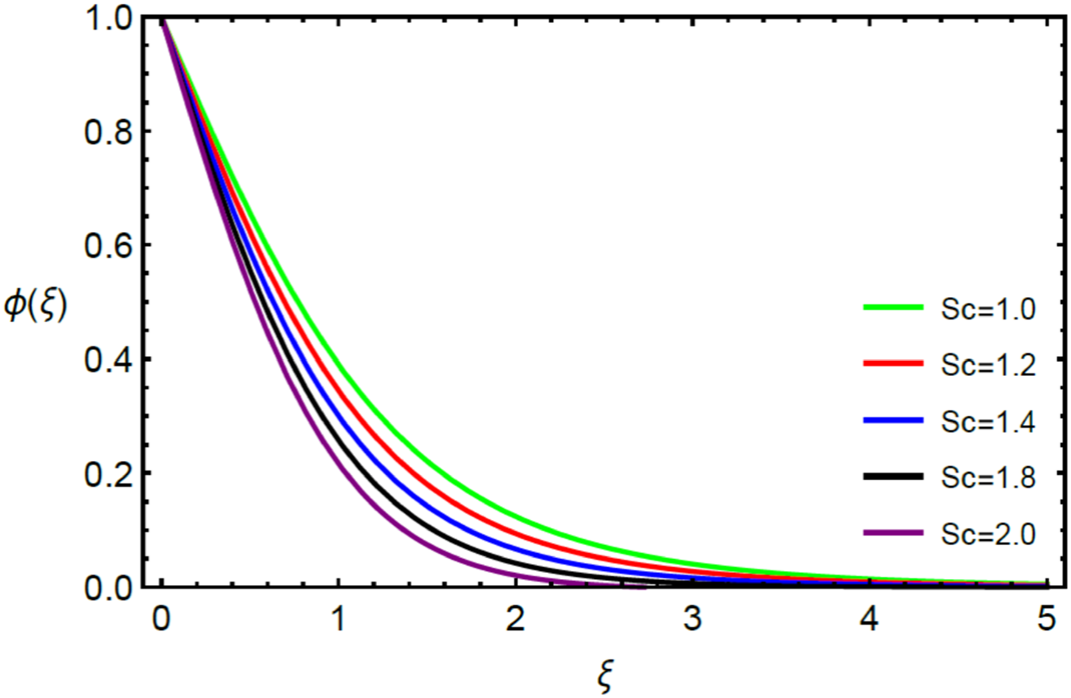
Inspiration of $\left( {Sc} \right)$ on $\phi \left( \xi \right)$.

**Figure 14:**
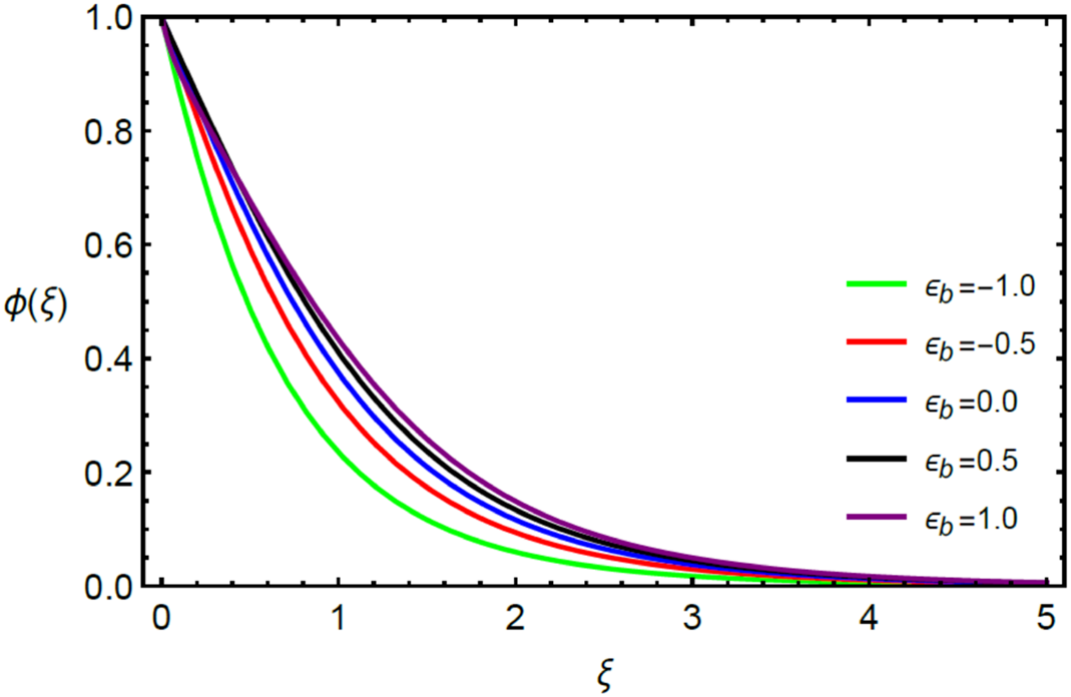
Influence of $\left( { \in_{b} } \right)$ on $\phi \left( \xi \right)$.

**Figure 15:**
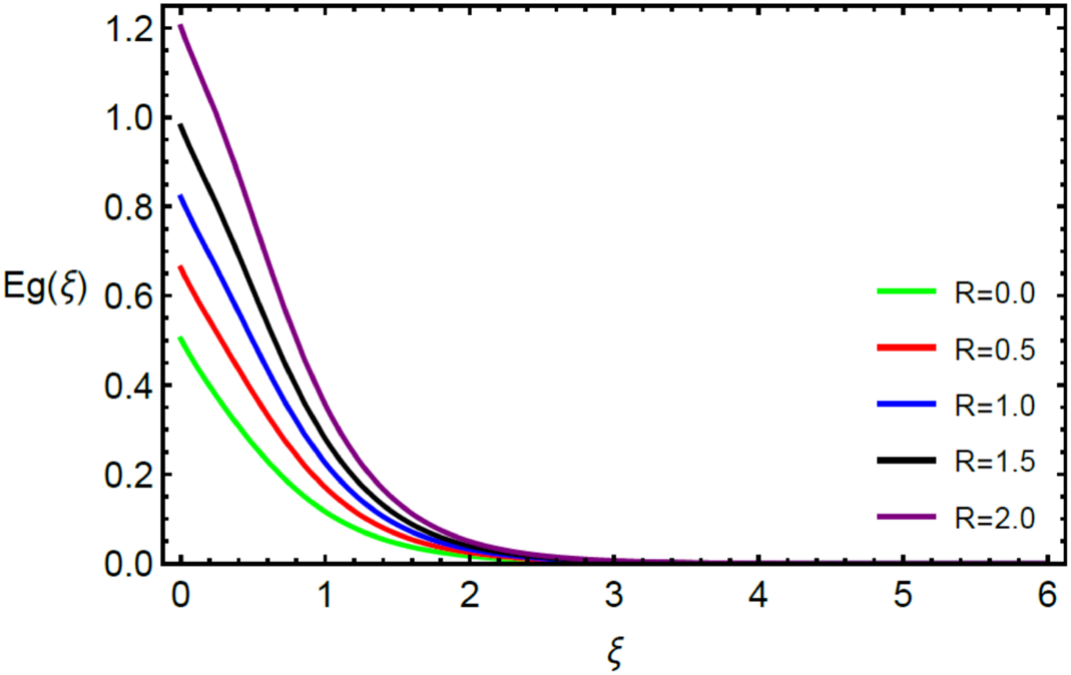
Influence of $\left( R \right)$ on $Eg\left( \xi \right)$.

**Figure 16:**
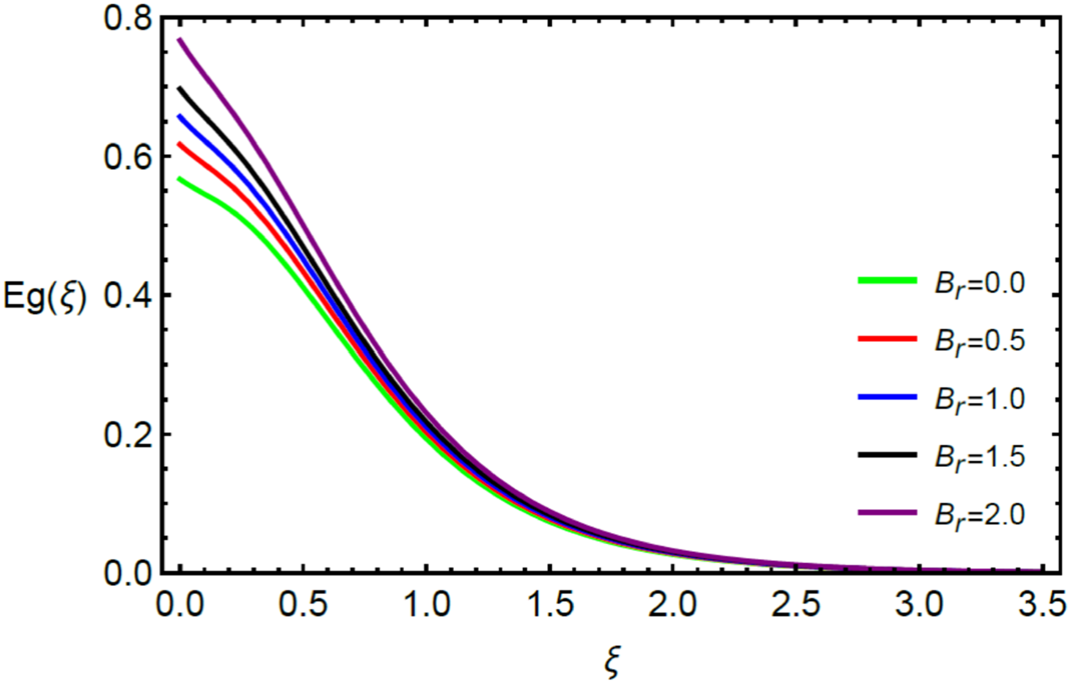
Influence of $\left( {B_{r} } \right)$ on $Eg\left( \xi \right)$.

**Figure 17:**
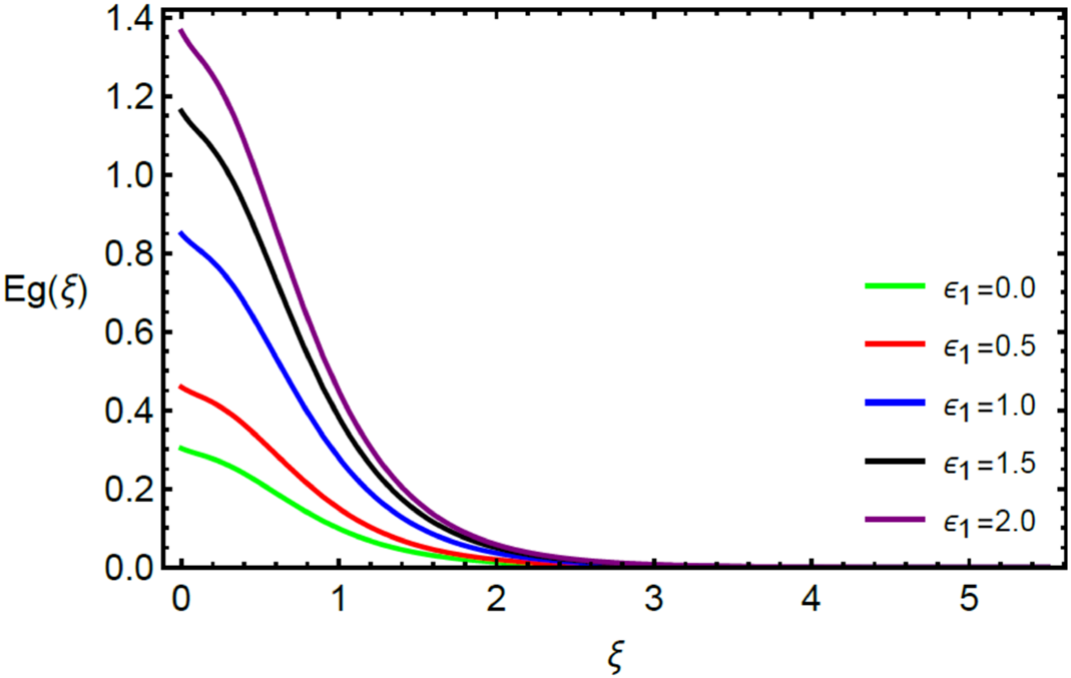
Influence of $\left( { \in_{1} } \right)$ on $Eg\left( \xi \right)$.

**Figure 18:**
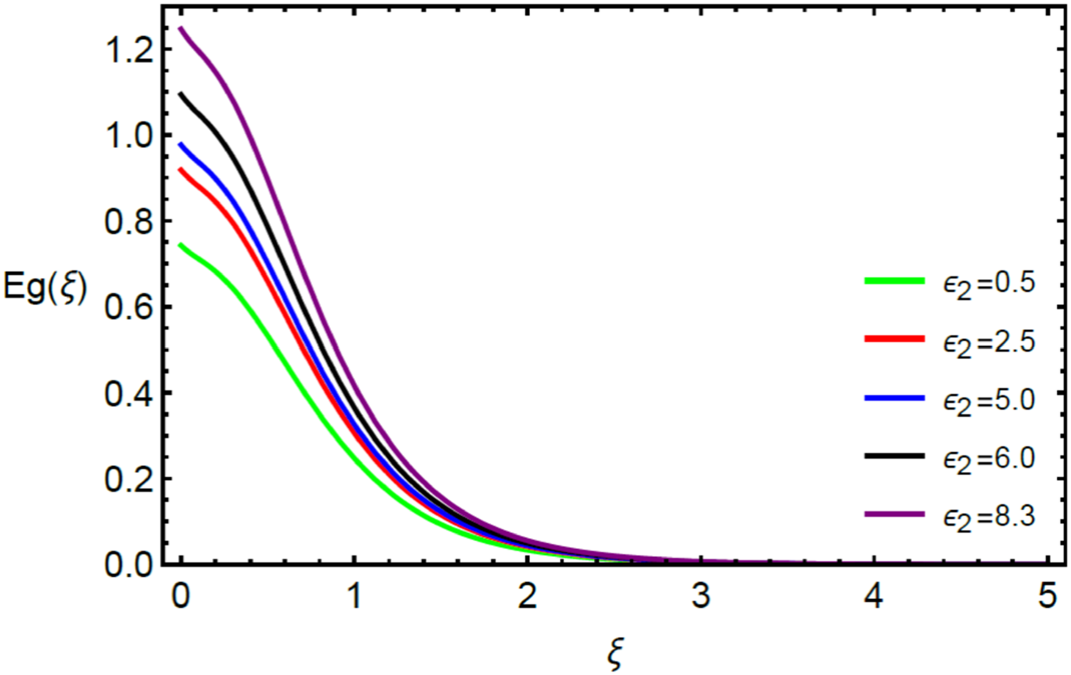
Influence of $\left( { \in_{2} } \right)$ on $Eg\left( \xi \right)$.

**Figure 19:**
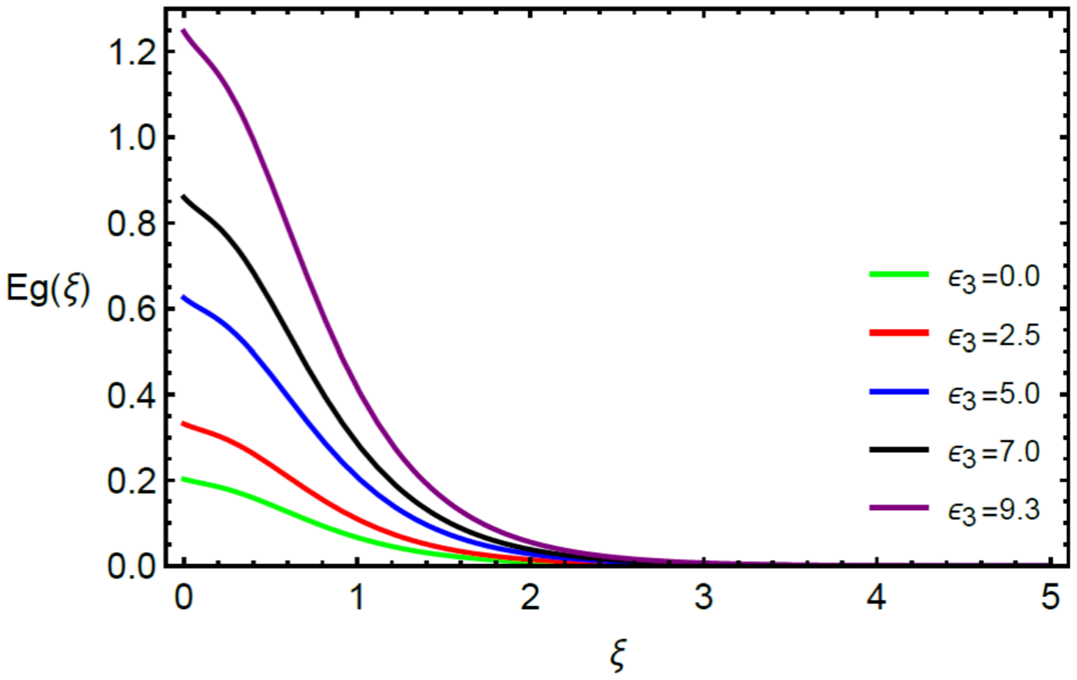
Influence of $\left( { \in_{3} } \right)$ on $Eg\left( \xi \right)$.

Figures 2 and 3 highlight slowed stream flow in $x_{1}$ and $x_{2}$ directions of the stretching surface as Casson fluid parameter $\left( \beta \right)$ is enhanced. This is due to the drop in yield stress at higher values of $\left( \beta \right)$ which causes the fluid to behave more like a Newtonian fluid, hence the velocities are seen to fall. Figures 4 and 5 shine light on the elevated thermal and mass profiles due to high $\left( \beta \right).$ Rise in $\left( \beta \right)$ causes elasticity stress parameter to intensify which in turn effects the temperature and concentration of the system positively. Figures 6, 7, 8 and 9 show the effects of increasing magnetic parameter $\left( {Ha} \right)$ on the velocity, thermal and mass behaviors. $\left( {Ha} \right)$ contributing directly to the resistive Lorentz force causes the velocities in both the directions to decay, as seen in Figs. 6 and 7, and due to the fall of fluid speed, the system is noted to heat up and become denser as represented in Figs. 8 and 9, respectively. Decline in velocity happens due to resistance during flow in associated region. Moreover, molecular vibration heightens the thermal and species transmission. Figure 10 focuses on the positive influence of radiation $\left( R \right)$ on the temperature of the system. This happens because higher $\left( R \right)$ results in the thicker thermal boundary layer which results to enhance temperature. Moreover, involvement of radiation improves conduction phenomenon which upturns the temperature field. Figure 11 shows a rise in temperature profile for incrementing values of $\left( {\varepsilon_{a} } \right).$ This is because rising $\left( {\varepsilon_{a} } \right)$ results in higher thermal conductivity and hence more heat is transferred from plate to liquid. In Fig. 12 the system is seen to cool down for raising values of Prandtl number $\left( {\Pr } \right)$ because of its inverse relation to thermal diffusivity. Against larger values of Prandtl number fluid temperature and associated boundary layer thickness reduces. Inverse bearing of thermal diffusion causes to lessen the thermal distribution. Similarly, due to the inverse relation of Schmidt number $\left( {Sc} \right)$ to the mass diffusivity, the concentration boundary layer becomes thinner for incrementing values of $\left( {Sc} \right)$ as noted in Fig. 13. Since Schmidt number has the inverse relation with mass diffusion. Incrimination in Schmidt number is due to lessen in mass diffusion which results to slow down the concentration field. Figure 14 displays the positive effect of $\left( {\varepsilon_{b} } \right)$ on mass boundary layer as with increasing $\left( {\varepsilon_{b} } \right)$ the mass diffusivity of the fluid improves and hence mass boundary layer thickens. Dimensionless entropy generation $Eg\left( \xi \right)$ is studied in Figs. 15, 16, 17, 18 and 19 against various parameters. In Figs. 15 and 16, $Eg\left( \xi \right)$ is noted to rise for increasing radiation parameter $\left( R \right)$ and Brinkman number $\left( {Br} \right).$ Since $\left( {Br} \right)$ and $\left( R \right)$ are contributions of Joule heating and thermal radiation, respectively, hence their rise causes more instability in the system. Rise in temperature difference parameter $\left( { \in_{1} } \right)$, concentration difference parameter $\left( { \in_{2} } \right)$ and the diffusion parameter $\left( { \in_{3} } \right)$ heighten the phenomenon of concentration irreversibility, which too is a direct contributing factor in the process of entropy generation, therefore $Eg\left( \xi \right)$ is noted to grow in Figs. 17, 18 and 19. Table 3 is constructed to validate the applied scheme in comparison with already published work for $- \left( {1 + \frac{1}{\beta }} \right)f^{\prime\prime}(0)$ and $- \left( {1 + \frac{1}{\beta }} \right)g^{\prime\prime}(0)$ taking $\beta = \infty , \;Ha = 0.$ A admirable arrangement is comprehended for the technique used in this exploration compared with the earlier published studies which use shooting method and MATLAB built in package bvp5c. Moreover, Table 4 is prepared to record the behavior of dimensionless stresses against the mounting values of ratio parameter by setting $\beta =\infty , Pr=0.7, Sc=0.5, Ha=0.$ Results are listed by comparing the obtained values of stresses with three different approaches. The recorded results shown an excellent settlement with those of reported in ref.^2^ and ref.^3^ in the absence of magnetic effect which retards the flow and control the turbulence. Also these results show an enhancement in dimensionless stresses against the fluctuating values of ratio parameter.

**Table 3:** Analysis of $- \left( {1 + \frac{1}{\beta }} \right)f^{\prime\prime}0)$ and $- \left( {1 + \frac{1}{\beta }} \right)g^{\prime\prime}(0)$ for $\beta =\infty , Ha=0.$

$n$	$\alpha$	$- \left( {1 + \frac{1}{\beta }} \right)f^{\prime\prime}0)$^4^		$- \left( {1 + \frac{1}{\beta }} \right)g^{\prime\prime}(0)$^4^		Present results using OHAM	
		Shooting	Bvp5c	Shooting	Bvp5c	$- \left( {1 + \frac{1}{\beta }} \right)f^{\prime\prime}0)$	$- \left( {1 + \frac{1}{\beta }} \right)g^{\prime\prime}(0)$
$1.0$	0	$1.0$	1.0	$0.0$	0.0	$1.0$	0.0
$-$	0.5	$1.224745$	1.224742	$0.612372$	0.612371	$1.224744$	$0.612371$
$-$	1.0	$1.414214$	1.414214	$1.414214$	1.414214	$1.414214$	1.414214

**Table 4:** Comparative studies for $- \left( {1 + \frac{1}{\beta }} \right)f^{\prime\prime}0)$ and $- \left( {1 + \frac{1}{\beta }} \right)g^{\prime\prime}(0)$ when $\beta = \infty , \;Pr = 0.7, \;Sc = 0.5, \;Ha = 0.$

$\alpha$	HPM^2^		Exact^2^		^3^	^3^	Present	Present
	− $f^{\prime\prime}(0)$	$g^{\prime\prime}(0)$	$f^{\prime\prime}(0)$	$g^{\prime\prime}(0)$	$f^{\prime\prime}(0)$	$g^{\prime\prime}(0)$	$f^{\prime\prime}(0)$	$g^{\prime\prime}(0)$
0.0	1.0	0.0	1.0	0.0	1.0	0.0	1.0	0.0
0.1	1.02025	0.06684	1.020259	0.066847	1.02026	0.06685	1.020257	0.066845
0.2	1.03949	0.14873	1.039495	0.148736	1.03949	0.14874	1.039489	0.148738
0.3	1.05795	0.24335	1.057940	0.243359	1.05795	0.24336	1.057938	0.243350
0.4	1.07578	0.34920	1.075788	0.349208	1.07578	0.34921	1.075785	0.349202
0.5	1.09309	0.46520	1.093095	0.465204	1.09309	0.46521	1.093073	0.465201
0.6	1.10994	0.59052	1.109946	0.590528	1.10994	0.59053	1.109942	0.590523
0.7	1.12639	0.72453	1.126397	0.724531	1.12639	0.72453	1.126395	0.724530
0.8	1.14248	0.86668	1.142488	0.866682	1.14249	0.86668	1.142487	0.866680
0.9	1.15825	1.01653	1.158253	1.016538	1.15826	1.016538	1.158250	1.016535
1.0	1.17372	1.17372	1.173720	1.173720	1.173720	1.17372	1.173720	1.173720

## Conclusions

Flow of Casson model under boundary layer has been scrutinized over a linearly stretching surface immersed in porous medium. Temperature dependent conductivity and diffusivity has been taken into consideration. Using OHAM analytical solutions for momentum, thermal and mass boundary layers have been obtained and examined against various parameters. Error analysis reveals the effectiveness of utilized algorithm. Proposed procedure has the ability to handle the other nonlinear problems occurring in other discipline of science. Main verdicts of this study are presented as follows:

- Fluid velocity in the $x_{1}$ and $x_{2}$ directions reduces when values of Casson fluid $\left( \beta \right)$ and magnetic $\left( {Ha} \right)$ parameters are enhanced.
- The system heats up with elevating Casson fluid parameter $\left( \beta \right),$ magnetic parameter $\left( {Ha} \right),$ small scalar parameter $\left( {\varepsilon_{a} } \right)$ and radiation $\left( R \right)$ parameters while opposite impact is noticed for the mounting values of Prandtl number $\left( {\Pr } \right).$
- Thicker boundary layer is recorded against $\left( {\varepsilon_{a} } \right)$ and $\left( R \right).$
- Temperature and concentartion fields are enhanced for the mounting values of Hartman number, whereas, decline in the velocity field is observed.
- Fluid becomes dense for incrementing Casson fluid $\left( \beta \right),$ magnetic $\left( {Ha} \right)$ and small scalar parameter $\left( {\varepsilon_{b} } \right)$ parameters while thinned out for Schmidt number $\left( {Sc} \right).$
- The system’s stability at a molecular level is controlled by diminishing values of radiation $\left( R \right),$ temperature difference $\left( { \in_{1} } \right),$ concentration difference $\left( { \in_{2} } \right),$ diffusion parameters $\left( { \in_{3} } \right)$ and Brinkman number $\left( {Br} \right).$
- Thermal transmission is the growing function of $\left( \beta \right),$ magnetic parameter $\left( {Ha} \right),$ small scalar parameter $\left( {\varepsilon_{a} } \right)$ and radiation $\left( R \right).$
